# A Novel Prognostic Signature Revealed the Interaction of Immune Cells in Tumor Microenvironment Based on Single-Cell RNA Sequencing for Lung Adenocarcinoma

**DOI:** 10.1155/2022/6555810

**Published:** 2022-07-01

**Authors:** Xing Jin, Zhengyang Hu, Qihai Sui, Mengnan Zhao, Jiaqi Liang, Zhenyu Liao, Yuansheng Zheng, Hao Wang, Yu Shi

**Affiliations:** ^1^Department of Thoracic Surgery, Zhongshan Hospital, Fudan University, No. 180, Fenglin Road, Shanghai 200032, China; ^2^Department of Pancreatic Surgery, Shanghai Cancer Centre, Fudan University, Shanghai, China

## Abstract

**Background:**

The tumor immune microenvironment (TIME) played an important role in immunotherapy prognosis and treatment response. Immune cells constitute a large part of the tumor microenvironment and regulate tumor progression. Our research is dedicated to studying the infiltrating immune cell in lung adenocarcinoma (LUAD) and seeking potential targets.

**Methods:**

The scRNA-seq data were collected from our FDZSH and two public datasets. The code for cell-type mapping algorithms was downloaded from the CIBERSORTx portal. The bioinformatics data of LUAD patients could be approached from The Cancer Genome Atlas (TCGA) portal. Weighted gene coexpression network analysis (WGCNA) and least absolute shrinkage and selection operator (LASSO) analyses were performed to construct a risk model. TIMER2 and TIDE helped with the immune infiltration estimation, while PROGENy helped the cancer-related pathways' enrichment analysis. GSE31210 dataset and IMVigor ICB therapy cohort validated our findings as the external validation datasets.

**Results:**

We clustered the scRNA-seq dataset (integrating our FDZSH datasets and other public datasets) into 23 subpopulations. After curated cell annotation, we implemented Cibersort and WGCNA analysis to anchor the brown module and natural killer cell cluster1 due to the most relationship with tumor trait. The overlap of the brown module gene, natural killer cell signature, and DEGs of tumor and adjacent normal samples was screened by LASSO Cox regression. The obtained 5-gene risk model showed an excellent prognostic performance in the validation dataset. Furthermore, there was a correlation between risk score and tumor-infiltrating immune cells and tumor genomics abnormity. Patients with higher risk scores had a significantly lower immunotherapy response rate.

**Conclusion:**

Our observations implied that immune cells played a pivotal role in TIME and established a 5-gene signature (including IDH2, ADRB2, SFTPC, CCDC69, and CCND2) on the basement of nature killer markers targeted by WGCNA analysis. The significance of clinical outcome and immunotherapy response prediction was validated robustly.

## 1. Introduction

Lung cancer is the most common cancer and the most prevalent cause of tumor-related death in the world [1]. Lung adenocarcinoma (LUAD) accounts for 85% of cases [2, 3]. Despite the significant advance in LUAD multidiscipline treatment, including surgery, chemotherapy, radiotherapy, and especially targeted therapy, the five-year survival rate of patients with LUAD remains discouragingly low. As the merging therapy, immunotherapy holds tremendous promise in controlling or even eradicating residual disease and improving cancer treatment and prognosis [4], but many patients still do not respond to anti-PD-1/PD-L1 immunotherapy. The current opinion is that the reciprocal regulation between tumor cells and tumor-infiltrating immune cells shapes the immune status of the TME [5] and may determine the outcome of cancer progression. Hence, the comprehensive analysis of the immune cells in LUAD patients facilitates the exploitation of novel biomarkers to predict the treatment response and disease prognosis.

With the development of sequencing technology over the past decade, molecular prognostic markers of tumors based on RNAseq technology emerged in endlessly. As a hot new technology in transcriptional analysis, scRNA-seq technology enables single-cell sequencing technology to reveal cellular gene expression that cannot be detected by bulk RNA sequencing [6]. Single-cell sequencing is mostly used to identify cell subgroups and pedigree analysis initially. With the maturity of scRNA-seq, single-cell sequencing technology in analyzing the tumor microenvironment is starting to become mainstream in oncology research. Currently, the exploration and in-depth analysis of scRNA-seq data of tumor specificity are still of great significance for the mass use of bulk RNA sequencing to characterize different cell subsets for using bulk RNA sequencing to characterize different cells subsets in large quantities.

This study integrated scRNA-seq data from our hospital and two external public databases with curated cell identity annotations. Furthermore, weighted gene coexpression network analysis (WGCNA) was implanted on the normal and tumor samples of the TCGA-LUAD cohort to explore the key module and cluster associated with tumor status. Consequently, the hub gene was selected to construct a five-gene risk model by the least absolute shrinkage and selection operator (LASSO) Cox regression algorithm and AUC validation. Our downstream analysis proclaimed the prospect and functionality of the signature in immune infiltration, mutational status, oncogenic pathways, and clinical prognosis; our results provide new sight into the immune cells in tumor heterogeneity and biomarker mining.

## 2. Materials and Methods

### 2.1. Single-Cell RNA Sequencing Data Collection

Fourteen primary LUAD patients who had received surgical resection in the Department of Thoracic Surgery in Zhongshan Hospital (FDZSH) were included for scRNA sequencing [7]. The diagnosis of lung adenocarcinoma was confirmed in each case by histopathological analysis. The other two public datasets, two independent LUAD patient cohorts, were downloaded from ArraryExpress (accession numbers E-MTAB-6149 and E-MTAB-6653) and Human Cell Atlas Data Coordination Platform (accession number PRJEB31843).

### 2.2. The Process of scRNA Dataset Integration and Cell Annotation

Preparation for single-cell transcriptomic sequencing followed the protocol for the 10x Genomics Chromium Single-Cell platform. Our previous published literature described the detailed tissue processing, and the single-cell suspension was described in our previous published literature [7].

We followed the Seurat v3 guidelines for the routine procedure. Cells expressing less than 200 genes or greater than 7000 genes or more than 20% mitochondrial genes were removed in the cell QC procedure. After normalization and PCA dimension reduction, the harmony [8] R package was utilized for removing the batch effect. Cell clustering was based on PCA dimensionality reduction using the first 20 PCs and a resolution value of 0.4. Marker genes manually identified cell annotation in the CellMarker database (http://biocc.hrbmu.edu.cn/CellMarker/) with the assistance of SingleR [9] and scType [10]. The marker genes of each cluster were conducted by the function FindAllMarkers() with the default parameters.

### 2.3. The TCGA Data Collection and Analysis

The bulk RNA sequencing (RNA-seq), genomics data, and clinical features of The Cancer Genome Atlas (TCGA)–LUAD tumor and adjacent normal samples were collected from the UCSC Xena (https://gdc.xenahubs.net). The differential gene analysis between the 56 paired tumor and normal sample of TCGA-LUAD was performed using the R package limma [11] with the threshold (FDR < 0.05 and Log (Foldchange) > 1).

### 2.4. The CIBERSORTx and WGCNA Analysis

The cell type abundance of TCGA samples from our scRNA-seq annotation was calculated by CIBERSORTx [12] using our customized signature matrix. The customized cell-type-specific signature genes were created by the creation feature of CIBERSORTx (https://cibersortx.stanford.edu). WGCNA was performed using the R package WGCNA [13] (version 1.70). Firstly, we chose both normal and tumor TCGA-LUAD samples for the analysis; the outlier sample was removed by setting the cutHeight 140. The soft power was determined by the pickSoftThreshold() function and setting the networkTYPE = “unsigned.” Power of 5 was chosen. We set WGCNA “mergeCutHeight” to 0.25 and merged the similar small module to identify nine modules. Of the nine WGCNA modules, the gray module includes genes that do not coexpress and are unassigned to a coexpression network; therefore, the gray module was excluded from our analysis.

### 2.5. LASSO Algorithm Model Construction

LASSO is a widely used regression method appropriate for analyzing data with high dimensions and strong relationships like high-throughput data. We used the R package glmnet to perform the LASSO Cox regression analysis as previously described [14], including a built-in crossvalidation function to adjust the L1 regularization variable lambda for candidate feature selection. The R caret package (https://cran.r-project.org/package=caret) was applied to build the classification of the TCGA-LUAD cohort and assess machine learning classifiers for the classification task.

### 2.6. Immune Infiltration Estimation and Genomic Analysis

The immune infiltration and cell composition estimation of TCGA patients were based on the TIDE (http://tide.musc.edu/) and TIMER2 (http://timer.cistrome.org/). The MAF file was used to illustrate the distribution of mutation frequency and status by the R package maftools [15]. Tumor mutational burden (TMB) was calculated as the number of somatic base substitutions or indels per megabase (Mb) of the coding region target territory of the test (currently, 1.11 Mb). The stemness data was from previous researchers' articles [14, 16, 17].

### 2.7. Statistical Analysis

The statistical analysis was conducted using R4.2.0. The Log-rank survival analysis and univariate and multivariate Cox proportional hazards regression by the stepwise method were performed using “survival” and “survminer.” The nomogram construction, validation, and calibration were performed and plotted using “rms” and “Hmisc” packages. The statistical analysis was performed via unpaired Student's *t*-test analysis or Wilcoxon signed rank test unless otherwise specified. All *p* values were two-sided, and *p* < 0.05 indicated statistical significance (∗*p* value < 0.05, ^∗∗^*p* value < 0.01, ^∗∗∗^*p* value < 0.001).

## 3. Results and Discussion

### 3.1. The Identification of Cell Type Based on the Integrated scRNA-Seq Datasets

**Figure 1:**
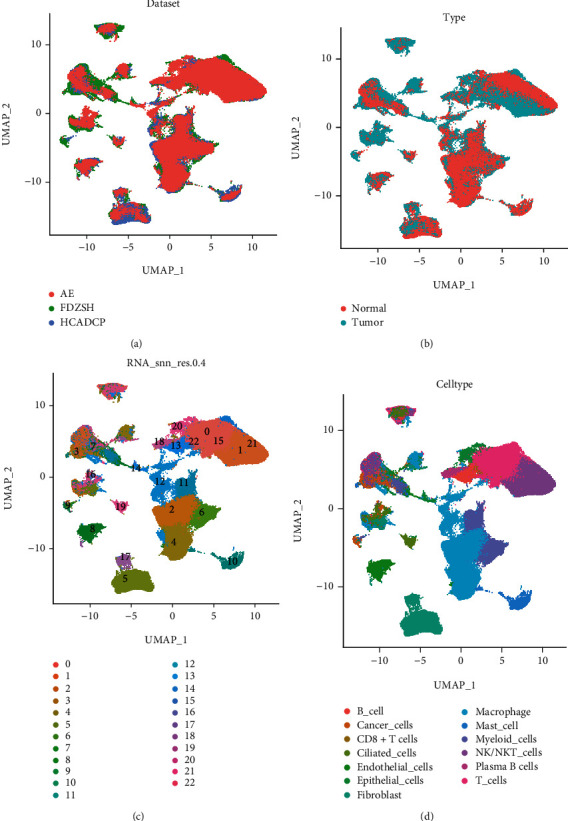
The landscape of sc-RNA seq cluster and annotation. (a) The Umap plot of three derived datasets of our analysis. (b) The normal and tumor type of cells. (c) The original Umap plot of the 24 clusters. (d) The cell annotation of the clusters.

First, we performed the routine quality control and normalization before integrating three datasets' scRNA-seq data by harmony algorithm. Figures 1(a) and 1(b) show the panoramic integration of three datasets across normal and tumor samples, demonstrating the robustness of our batch integration. After performing the PCA and UMAP dimension reduction, we identified 23 clusters. Using the *FindAllMarkers()* function (Threshold: LogFC:1), we defined the signature genes of each cluster. Each cluster's label was firstly annotated by the SingleR and scType automatically. The final curated annotation was completed via the CellMarker database. The detailed information on cell cluster identification was demonstrated in Table S1.

**Figure 2:**
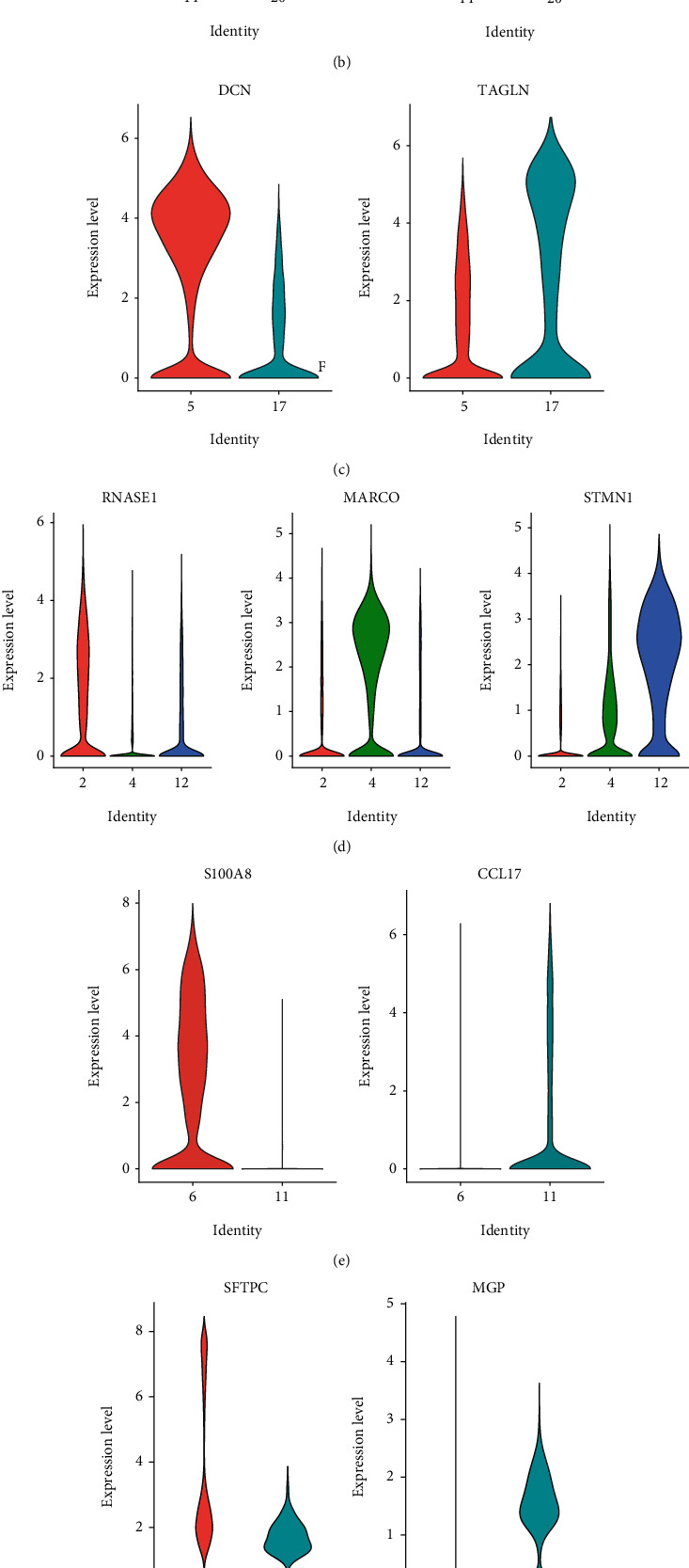
The cell annotation and the specially expressed genes in subgroups. The conserved expressed marker genes of cancer cells (a), epithelial cells (b), fibroblast (c), macrophages (d), myeloid cells (e), nature killer cells (f), and T cells (g).

During the cell annotation, universal marker genes classified the main cell type shown in the dotplot in Figure S1. Some subgroups of cell clusters were also defined. As four subclusters of cancer cells, clusters 3, 7, 9, and 16 expressed specially SCGB3A1, TMC5, PCP4, and AKR1C1 genes. Cluster 14 especially expressed TOP2A, indicating the proliferation potential of the epithelial cells. Cluster 5 highly expressed the DCN, the main fibroblast marker gene. The macrophages (Figure 2(d)), myeloid cells (Figure 2(e)), NK cell (Figure 2(f)), and T cell (Figure 2(g)) also had subgroups illustrated in Figure 2. These subpopulations provide unique transcriptional signatures that prompt us to investigate the functional heterogeneity in further analysis.

### 3.2. The Anchoring of the Key Module and Genes by WGCNA Analysis

**Figure 3:**
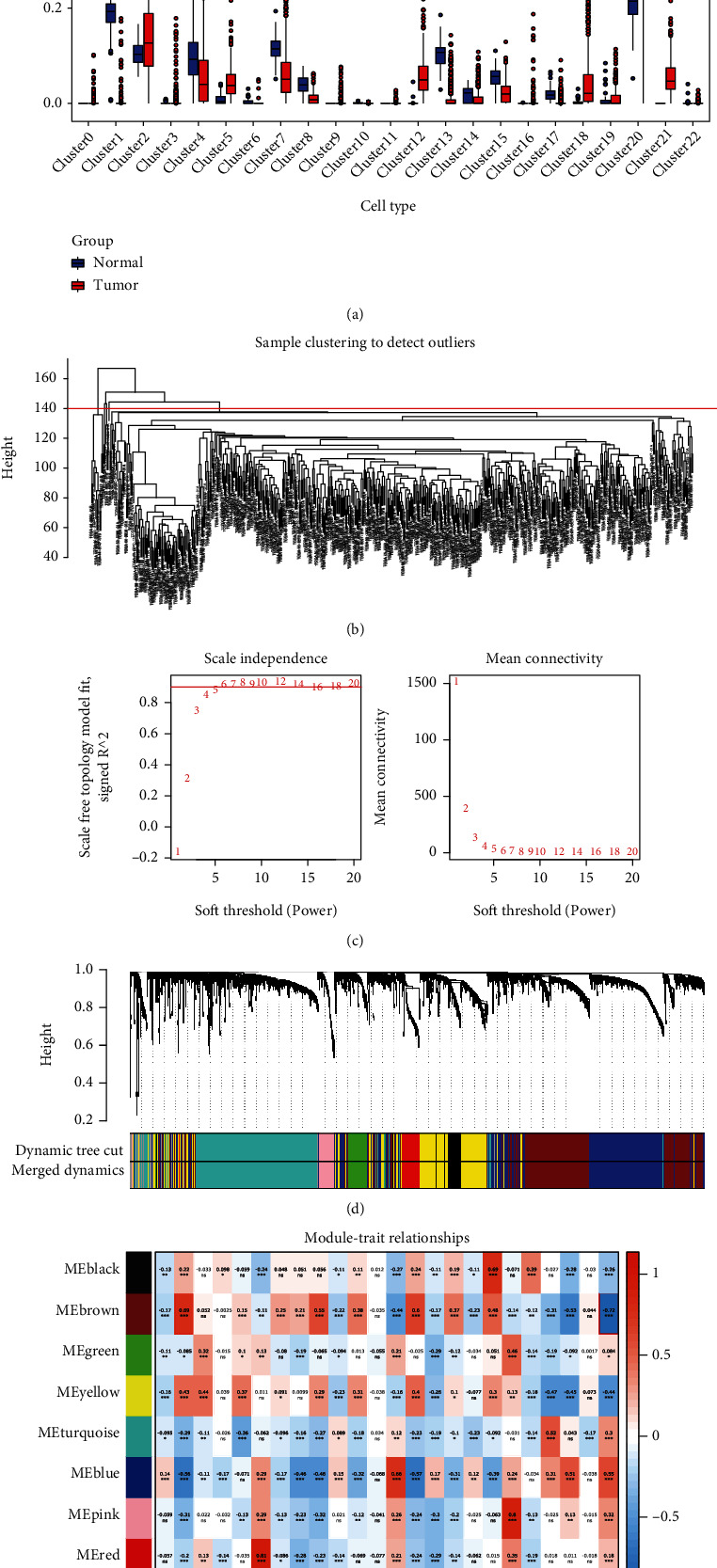
The mining of the hub module in WGCNA analysis. Determination of key module and key cluster in LUAD. (a) The cell abundance of the 23 clusters in bulk RNA seq of the TCGA-LUAD normal and tumor samples. (b) The sample clustering plot to remove outlier samples. (c) The scale-free fit index for various soft-thresholding powers. (d) dendrogram based on different metrics before and after merging small modules. (e) The nine modules, with the number in parentheses, represent the transcript number. Each row corresponds to a module, and each column corresponds to a cluster cell abundance. The color scale on the right shows module-trait correlations from 1 (red) to −1 (blue). Each cell at the row-column intersection's background color represents the correlation coefficient between the modules and clusters. The red color indicates a high degree of positive correlation, and the blue indicates a high degree of negative correlation between each module and the clusters. Each cell also contains the corresponding *p* signatures (bottom symbols).

Under the UMAP dimension reduction and cell-type annotation of our datasets, we formulated the CIBERSOT progress to calculate the cell abundance based on the transcriptome profile of our scRNA-seq. Considering the heterogeneity of each cluster, we included all the clusters rather than cell types to establish the signatures. The cell abundance of tumor and normal samples in the TCGA-LUAD database showed significant differences among 21 cell subpopulations (Figure 3(a)) except for myeloid cells and epithelial cells (clusters 11 and 16). The cell abundance of effector immune cells like NK cell (cluster 1), B cell (cluster 13), and T cell (cluster 15) in the tumor are lower than in paratumor tissue. Other immune cells like macrophages and myeloid cells showed heterogeneity among subclusters.

WGCNA analysis was performed to determine the correlation between gene expression module and cell abundance of specific cell subpopulations. All 585 TCGA-LUAD samples mingled with 59 adjacent and 526 tumor samples. The hierarchical clustering results and deleted outlier sample are shown in Figure 3(b).  Figure 3(c) showed that the soft threshold selection process and a scale-free network were successfully conducted (Figure S2). Finally, we obtained nine modules after merging small modules (Figure 3(d)). The correlation analysis between WGCNA modules and tumor phenotype showed that the brown module was most likely relevant to the tumor. Correspondingly, the closest cell cluster was the NK cell (cluster 1). Further investigation of the brown module genes was conducted. The GO and KEGG enrichment results are shown in Table S2 and Figure S2.

### 3.3. Construction and Validation of the Prognostic Value of the Risk Model

**Figure 4:**
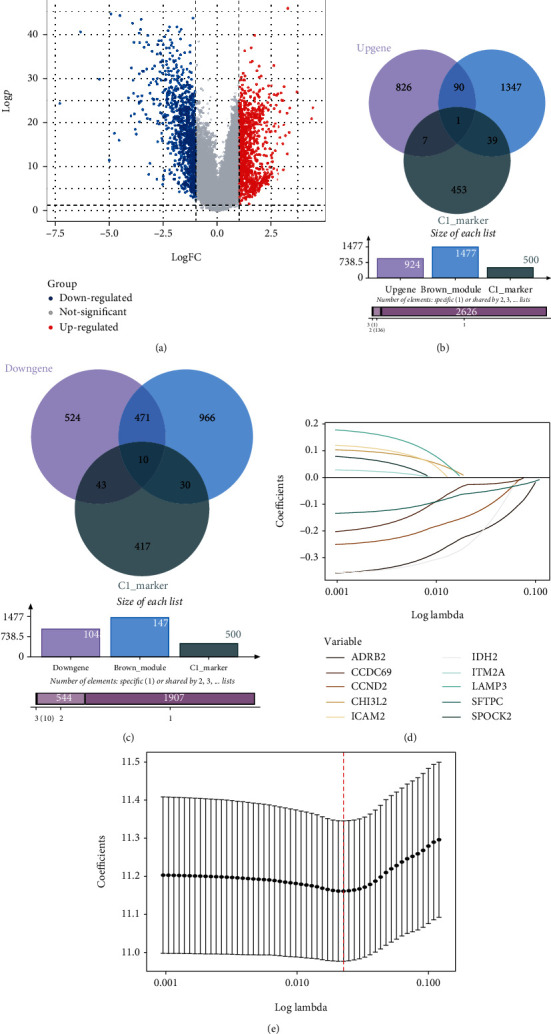
The construction of the risk model. The volcano plot of DEGs in the TCGA-LUAD cohort (a). The Venn plot identified the two overlap analyses of upregulated DEGs(b)/downregulated DEGs(c), brown module genes, and NK cell cluster1 signature genes. (d) The LASSO coefficient profile plot shows the correlation between the deviance and log(*λ*). (e) The partial likelihood of deviance for the LASSO Cox regression analysis.

Since the brown module and NK cell cluster 1 were identified in the WGCNA analysis, the further differential analysis on the expression profile of 56 normal/tumor paired samples in the TCGA-LUAD cohort. With the threshold *p* adjust value < 0.05 and logFC > 1, we obtained 1972 differential expressed genes (DEGs), of which 1048 genes were upregulated and 1048 were downregulated (Figure 4(a)). Venn diagram (Figures 4(b) and 4(c)) shows the intersected genes across the up/downregulated DEGs, NK cell cluster marker, and brown module's genes. The LASSO Cox model was conducted to build a prognostic model. Figures 4(d) and 4(e) show the selection of optimal lambda parameters for the LASSO model. The module formula described as follows: RiskScore = −0.213∗ADRB2 + −0.241∗IDH2 + −0.058∗SFTPC+−0.027∗CCDC69+−0.138∗CCND2. The expression level of genes was calculated and normalized to Log2 (FPKM + 1).

**Figure 5:**
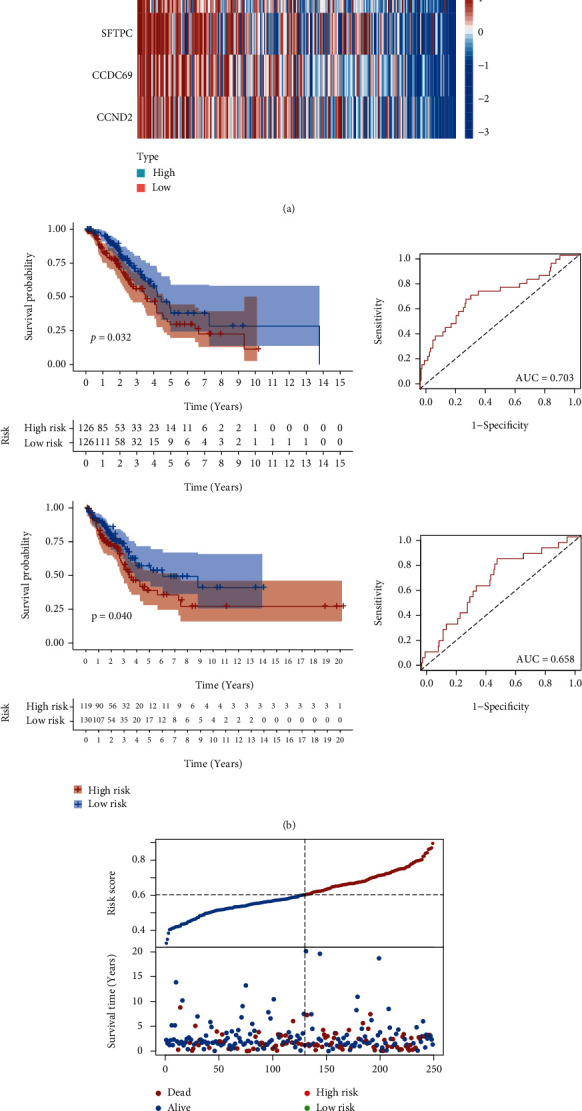
The training and validation of the risk model. The distribution of risk scores and survival status (up) and the gene expression heatmap (down) in training (a) and validating (c) dataset. (b) The survival plot (left) of the high-risk score group vs. low-risk group and ROC curve plot (right) in the train and validate dataset.

The validation of our model was based on the TCGA-LUAD cohort. The whole cohort was divided into the train and validated dataset based on the recursive feature elimination classification algorithm, often used in machine learning. Figures 5(a) and 5(c) show the risk score distribution of two datasets. The overall survival plot showed that patients with high-risk scores are more likely to have a poor prognosis. The area under this model's time-dependent ROC curve (AUC) to predict the 12-month survival in the train and validation dataset was 0.703 and 0.658. Considering the above evidence, our model has a robust and accurate prediction for the prognosis.

### 3.4. The Immune Landscape of Different Risk Groups in LUAD

**Figure 6:**
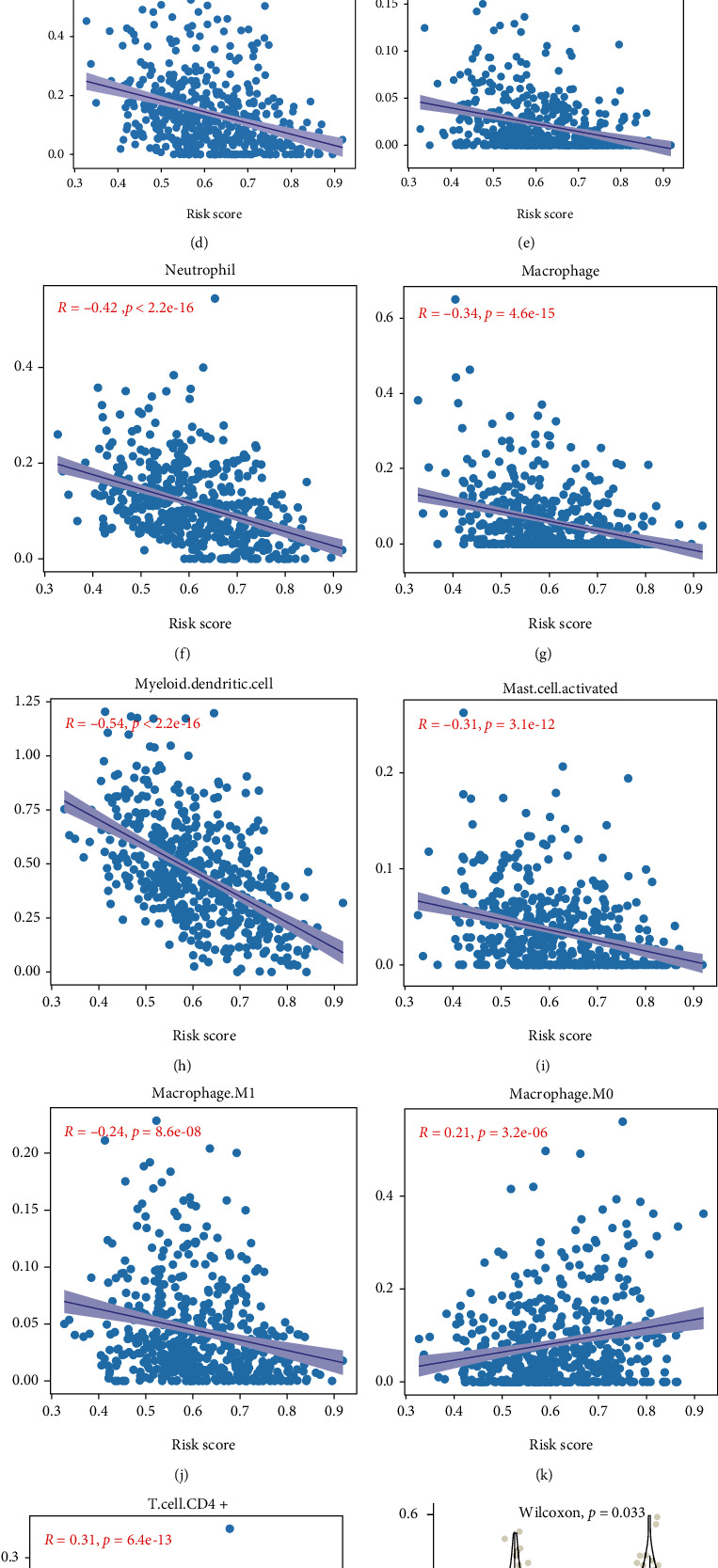
The correction between immune infiltration and risk scores. (a) The estimation of ImmuneScore and Tumorpurity by ESTIMATE in low- and high-risk score group. (b) Comparison of stemness in low- and high-risk score group; (c–l) the correlation analysis between the immune cells' abundance and risk score in TIMER2 with the threshold of *p* < 0.05 and *R*^2^ > 0.2. (m) Significant difference in TIDE score between the low- and high-risk score group. (n) The bar plot shows the percentage of response rate to immune treatment in low- and high-risk groups (green: response; red: no response).

The immune cells in the tumor microenvironment play an essential role in tumor prognosis. The association between the risk score and infiltration of immune cells was explored. The ESTIMATE formula computed the ImmuneScore and TumorPurity. Corresponding to the above result, the high risk score tumors had statistically significantly lower levels of immune infiltration (Figure 6(a)) (ImmuneScore: high vs. low: 1204 vs. 1812, *p* < 0.001) and higher tumor infiltration (TumorPurity: 0.692 vs. 0.599, *p* < 0.001). Additionally, the mRNAsi stemness score in high group was higher than low group (Figure 6(b)) (0.350 vs. 0.302, *p* < 0.001). The correlation analysis between the immune cell abundance and a risk score is shown in Figures 6(c)–6(l). The risk score was negatively correlated with the abundance of CD8+ T cell, NK cell, neutrophil cell, macrophage M1, myeloid dendritic cell, and memory B cell. CD4+ T cell and macrophage M0 positively correlate with a risk score. The TIDE prediction score in the low-risk group was significantly lower than in the high-risk group, representing the positive correlation between risk score and tumor immune escape (Figure 6(m)). The barplot in Figure 6(n) shows the predicted response of immunotherapy in the low and high groups corresponding to the abovementioned results. A conclusion could be inferred from the above results that the risk model could participate in regulating tumor microenvironment via immune cell infiltration and had a predictive value in immunotherapy response.

### 3.5. The Correlation between Risk Score and Genomic Features

**Figure 7:**
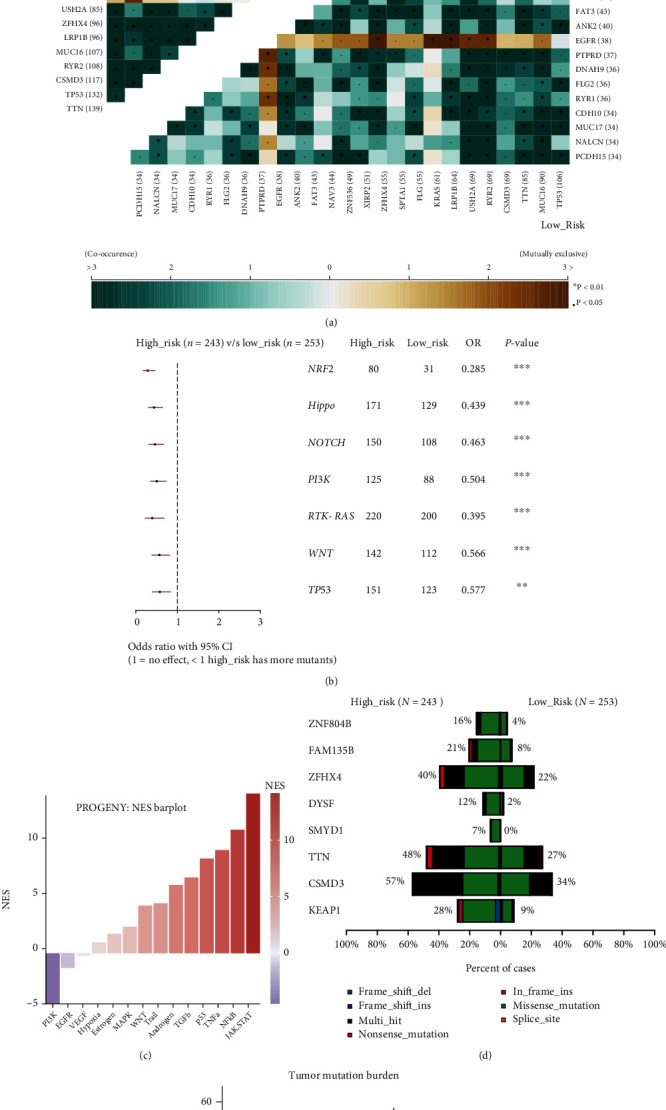
Genomic abnormities in different risk groups. (a) The high-risk (top left) and low-risk groups (bottom right) have different somatic interaction patterns. (b) Forest plot of the differential pathways between low- and high-risk groups. (c) The PROGENy pathway activity enrichment score for 11 cancer-associated signaling pathways (high-risk group vs. low-risk group). (d) Barplot between the low- and high-risk score group shows the top differential mutated genes. (e) Violin plot of tumor mutation burden between the two groups.

The investigation of the genomic features was conducted to reveal the tumor characteristics in TIME. The distribution of variants and somatic interactions of the low- and high-risk groups was shown in Figure 7(a) and Figure S3. The commonly driven genes like EGFR, KRAS, and KEAP1 mutated in a mutually exclusive manner. The differentially mutated oncopathways between low- and high-risk groups are shown in a forest plot in Figure 7(b). The pROGENY analysis also revealed that many oncopathways were enriched in the high-risk group. Drive gene KEAP1 has a significantly higher mutation frequency in the high-risk group (28% vs. 9%, *p* < 0.001). Consistent with the analysis above, the patients in the high-risk group hold an elevated tumor mutation burden (6.13 vs. 4.58, *p* < 0.001). The above results suggested that this model revealed that immune cell infiltration in the TIME affects the tumor's genomics status and mutation load.

### 3.6. The Analysis of Clinical Characteristics and Construction of Nomogram

**Figure 8:**
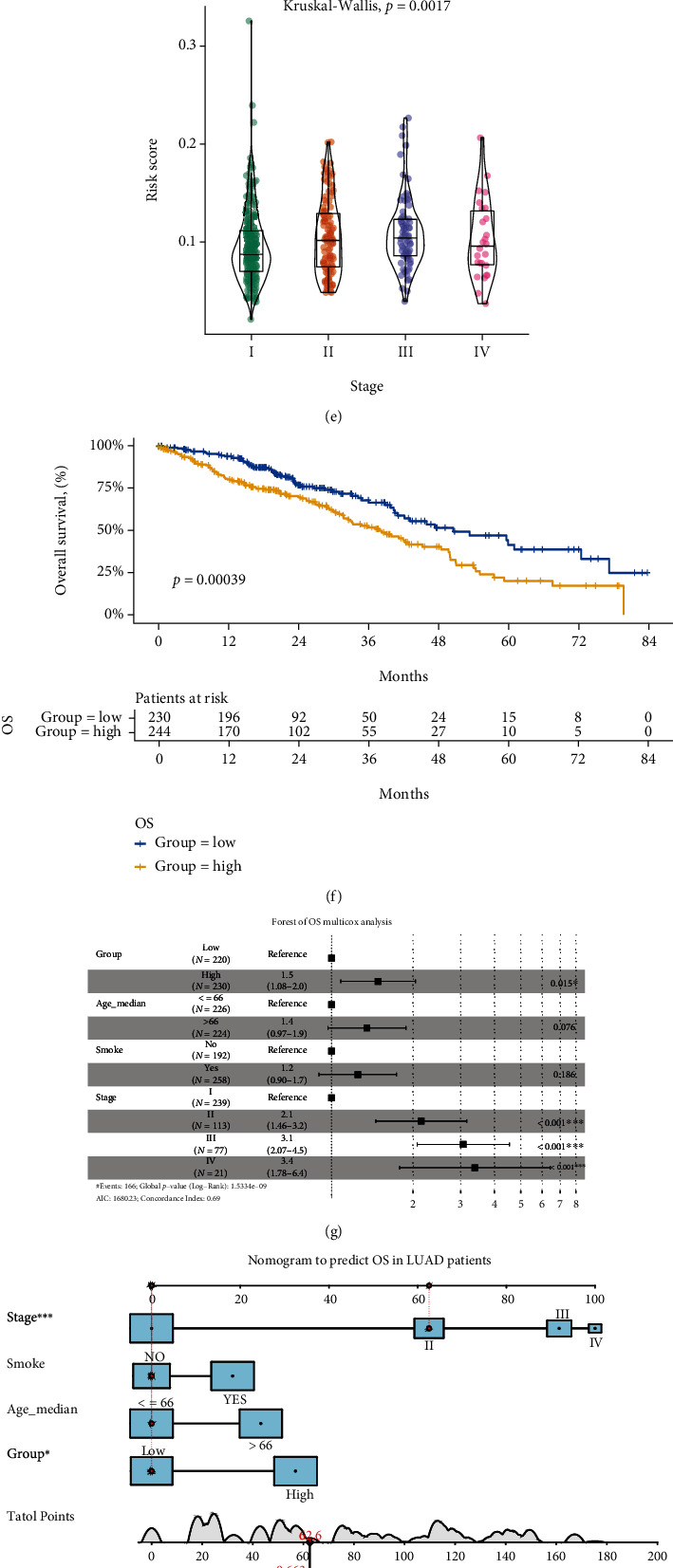
Clinical application of the 5-gene model in LUAD. (a–e) The difference in risk score by age group, sex, race, smoke, and stage. (f) The patients in the high-risk score group had significantly poor OS. (g) The forest plot shows the multivariate Cox regression analysis. (h) The nomogram was constructed by the risk group and other independent prognostic factors. (i) The calibration curves of the nomogram.

As Table S3 and Figures 8(a)–8(e) show, the risk score has no connection to the age, but the base characteristics between the two groups are not balanced. The overall survival (OS) results revealed that patients with lower risk scores exhibited better survival prognoses (Figure 8(f)). The median time of survival in the low-risk score group was 50.5 (95% CI: 40.9-NA) months, whereas high-risk score patients had a considerably shorter median survival time (37.83 [32.5, 48.4] months, *p* < 0.0001). Due to the baseline imbalance and confounding factors. We identified that the risk score group was a crucial independent prognostic factor by univariate and multivariate Cox regression analysis (Table S3, Figure 8(g)). The nomogram was established based on the significant factors, including group, age median group, smoke, and pathologic stage (C-index: 0.687). The validation of the nomogram was implemented through 1-, 3-, and 5-year calibration curve plots (Figure 8(i)), which demonstrated that our nomogram model performed well on the robustness and efficacy.

### 3.7. The External Validation in Prognostic Value and Potential to Predict Response Rate of Immunotherapy

**Figure 9:**
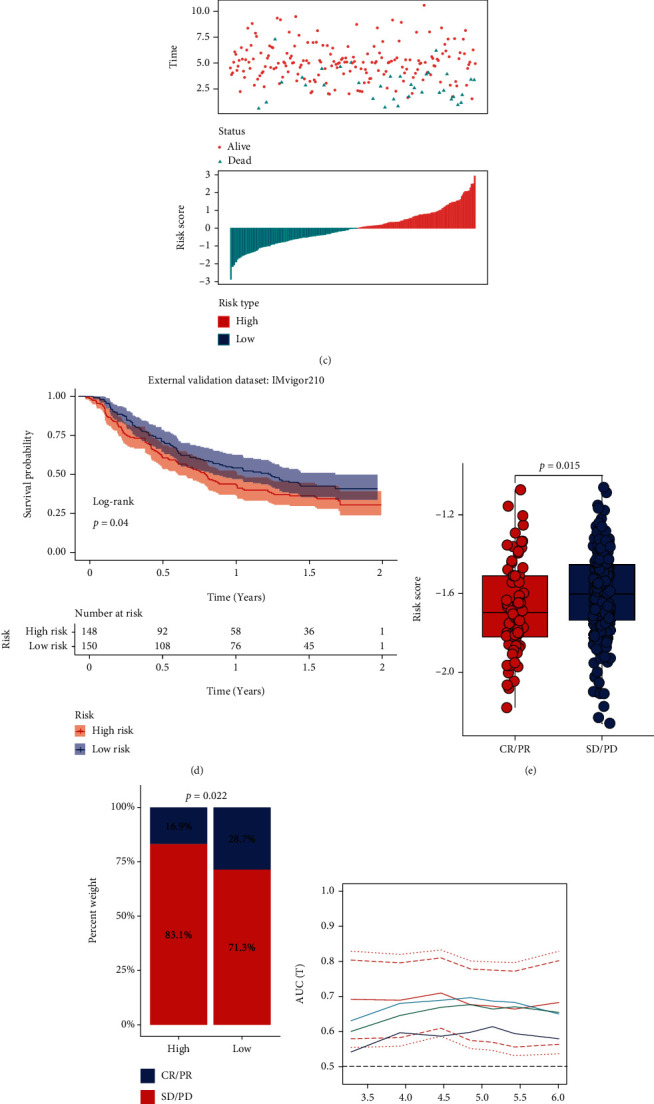
External validation and comparison with other signatures. (a–c) The external validation of our 5-gene model's prognostic value in GSE31210 dataset and the potential predictivity of immunotherapy response in IMVigor cohort (d–f). The AUC curve plot with pointwise confidence intervals (g) and ROC curve plot (h) of our signature and other researchers' signature for comparison.

To verify the robustness of our findings, we performed two further validation analyses. First, we used the independent external GSE31210 LUAD dataset to validate the prognostic value of our 5-gene model. The AUC value at one-, three-, and five-year time point is 0.59, 0.7, and 0.68 (Figures 9(a)–9(c)). Patients accepted ICB treatment in high-risk score group showed a significantly poorer outcome and a lower response rate in IMVigor 210 cohort (Figures 9(d)–9(f)). Moreover, we also collected other three immuned related signature models [18–20], the comparison of the four model was conducted in the independent GEOdataset because the training dataset of four models was TCGA-LUAD cohort; Figures 9(g) and 9(h) showed that the AUC value of our model was significantly higher than other models in various time points.

## 4. Discussion

Our research merged multiple single-cell datasets, annotated the main cell type, and identified their cluster-specific marker genes. A five-gene risk model was obtained by the NK cell cluster marker gene screened in WGCNA analysis due to the closest relationship to tumor traits. Subsequent analysis validated the independent predicted value and well performance in immunotherapy response and revealed the crucial role of immune cells within the TME in tumor progression and metastasis.

Since the rapid development and accessibility of scRNA-seq in cancer research, promising findings in cancer evolution, metastasis, and TME have been reached [21, 22]. Previous studies have demonstrated that single-cell transcriptome analysis could apply specific signature genes to estimate cell type abundances of bulk transcriptome [23]. Schaum et al. [24] performed the CIBERSORTx deconvolution algorithm on annotated scRNA-seq to quantify the abundance of immune cells in 17 organs at ten ages based on their massive bulk RNA seq data which confirmed her findings with scRNA-seq. Recent studies reported applying scRNA-seq and bulk RNA seq data to analyze the tumor heterogeneity and immune cells in ovarian cancer [25], glioma [26], and esophageal squamous cell carcinoma [27]. Jerby-Arnon et al. [28] identified a cancer cell-related T resistance program to predict the immunotherapy response in melanoma patients. Here, we merged our dataset with two other independent datasets to expand the applicability of our signature in LUAD and targeted the NK cell cluster 1 as the essential signature by the linkage of CIBERSORTx and WGCNA analysis. Interestingly, the cluster 1 special expressed gene SFTPC was identified as one of the 5-gene risk models, demonstrating the findings' sturdiness. Altogether, the development of scRNA-seq data promoted the investigation of novel biomarkers in the specific cancer type.

Our research also showed that immune cells are TIME's backbone in LUAD. In our silicon analysis, NK cell signature and its subsequent LASSO selection constructed the 5-gene risk model, which had a significant negative correlation with the NK cell abundance. We inferred that the risk score represents the exclusion of NK cells. A previous study illustrated that NK cells were lower in NSCLC than in noncancerous lung tissue [29], holding the bridge of innate and adaptive immune responses via interaction with other immune cells [30–32]. However, cancer cells could utilize immune escape mechanisms like expressing PD-L1 to impair the NK cell function in LUAD [33, 34].

On the contrary, NK cell infiltration could also generate durable and long-lasting antitumor immune responses against lung cancer. Our results showed that the lower risk score had a significantly higher immunotherapy response rate, a lower activated oncogenic pathways rate, and flat genomic abnormity. Generally, we suggested that our risk score inferred the infiltration of NK cells in the TIME of lung cancer.

Our research consequently identified the overlap of DEGs and NK cell cluster markers as the candidate for the risk model, and LASSO Cox regression helped us determine isocitrate dehydrogenase (NADP(+))2(IDH2), adrenoceptor beta 2(ADRB2), surfactant protein C (SFTPC), coiled-coil domain containing 69 (CCDC69), and cyclin D2 (CCND2). IDH2 is often considered to have a similar prognostic effect to IDH1 in glioma [35]. Li et al. [36] reported IDH2' as an indicator of poor prognosis and concluded that IDH2 promotes the Warburg effect and tumor proliferation through HIF1*α* in lung cancer. SFTPC encodes the pulmonary-associated surfactant protein C, a hydrophobic surfactant protein for maintaining stable pulmonary tissue. Moreno-Rubio et al. [37] reported the overexpression of SFTPC in long-term survival NSCLC patients, while a Norwegian group reported similar findings. They found that SFTPC and SFTPA mRNAs could be potential markers in regional nodes and peripheral blood in lung cancer [38]. In summary, we believe that the deep exploration of the molecular mechanism of the five gene model in TIME would facilitate the development of novel diagnostic biomarkers.

This study provides a new perspective on understanding the immune cells in TIME and sets a novel risk model; limitations to our research highlight the need for further work to optimize our work. Firstly, the internal validation of the model showed a good performance, and further external real-world validation is needed. Downstream and functional experiments underlying the mechanism of immune cells and model genes could help discover potential therapeutic targets. We plan to pursue applying the risk model to the diagnosis of early-rate LUAD.

## 5. Conclusions

Our study utilized the scRNA-seq data to identify the heterogeneous cell population in LUAD, applied the CIBERSORTx algorithm to map the cell type into the bulk RNAseq, revealed the key role of immune cells, especially natural killer cells, in TIME, and constructed the 5-gene model with the robust prognostic prediction and potential to evaluate immunotherapy response.

## Data Availability

Data adopted in this study are available in TCGA (http://portal.gdc.cancer.gov/) and UCSC Xena Browser (http://xena.ucsc.edu/). Other data are available from the corresponding authors upon reasonable request.

## Abbreviations

DEGs: Differential expression genes
GEO: Gene expression omnibus
LUAD: Lung adenocarcinoma
PPIs: Protein-protein interactions
RNA seq: RNA sequencing
scRNA-seq: Single-cell sequencing
TCGA: The Cancer Genome Atlas
TME: Tumor microenvironment
TIME: Tumor immune microenvironment
UMAP: Uniform manifold approximation and projection.

## Supplementary Materials

Supplementary Materials Figure S1: the cell annotation dot plot. The dot plot shows the percentage of clusters expressing marker genes (dot size) and the expression level (dot color). Figure S2: the GO and KEGG dot plot of brown module genes. The dot plot of KEGG (A) pathway and GO (B) enrichment terms. Figure S3: the summary and comparison of genomic analysis in the low- and high-risk groups. The low-risk (A) and high-risk groups (B) of patients' somatic mutation summary. The plots displayed the number of variants in each sample as a stacked bar plot and variant types as a boxplot summarized. Table S1: the cell annotation of 23 cell clusters. Table S2: the brown module's GO and KEGG enrichment analysis results. Table S3: the baseline characteristics between low- and high-risk groups.

## References

[1] Sung H., Ferlay J., Siegel R. L. (2021). Global cancer statistics 2020: GLOBOCAN estimates of incidence and mortality worldwide for 36 cancers in 185 countries. *CA: a Cancer Journal for Clinicians*.

[2] Gridelli C., Rossi A., Carbone D. P. (2015). Non-small-cell lung cancer. *Nature Reviews Disease Primers*.

[3] Bi G., Chen Z., Yang X. (2020). Identification and validation of tumor environment phenotypes in lung adenocarcinoma by integrative genome-scale analysis. *Cancer Immunology, Immunotherapy*.

[4] Couzin-Frankel J. (2013). Cancer immunotherapy. *Science*.

[5] Liu N., Luo J., Kuang D. (2019). Lactate inhibits ATP6V0d2 expression in tumor-associated macrophages to promote HIF-2*α*–mediated tumor progression. *Journal of Clinical Investigation*.

[6] Choi K., Raghupathy N., Churchill G. A. (2019). A Bayesian mixture model for the analysis of allelic expression in single cells. *Nature Communications*.

[7] Chen Z., Huang Y., Hu Z. (2021). Landscape and dynamics of single tumor and immune cells in early and advanced- stage lung adenocarcinoma. *Clinical and Translational Medicine*.

[8] Korsunsky I., Millard N., Fan J. (2019). Fast, sensitive and accurate integration of single-cell data with harmony. *Nature Methods*.

[9] Aran D., Looney A. P., Liu L. (2019). Reference-based analysis of lung single-cell sequencing reveals a transitional profibrotic macrophage. *Nature Immunology*.

[10] Choi J.-H., In Kim H., Woo H. G. (2020). scTyper: a comprehensive pipeline for the cell typing analysis of single-cell RNA-seq data. *BMC Bioinformatics*.

[11] Ritchie M. E., Phipson B., Wu D. (2015). Limma powers differential expression analyses for RNA-sequencing and microarray studies. *Nucleic Acids Research*.

[12] Li L., Shen L., Ma J. (2020). Evaluating distribution and prognostic value of new tumor-infiltrating lymphocytes in HCC based on a scRNA-seq study with CIBERSORTx. *Frontiers in Medicine*.

[13] Langfelder P., Horvath S. (2008). WGCNA: an R package for weighted correlation network analysis. *BMC Bioinformatics*.

[14] Jin X., Zheng Y., Chen Z. (2021). Integrated analysis of patients with KEAP1/NFE2L2/CUL3 mutations in lung adenocarcinomas. *Cancer Medicine*.

[15] Mayakonda A., Lin D.-C., Assenov Y., Plass C., Koeffler H. P. (2018). Maftools: efficient and comprehensive analysis of somatic variants in cancer. *Genome Research*.

[16] Malta T. M., Sokolov A., Gentles A. J. (2018). Machine learning identifies stemness features associated with oncogenic dedifferentiation. *Cell*.

[17] Zhao M., Li M., Chen Z. (2020). Identification of immune-related gene signature predicting survival in the tumor microenvironment of lung adenocarcinoma. *Immunogenetics*.

[18] Sun S., Guo W., Wang Z. (2020). Development and validation of an immune-related prognostic signature in lung adenocarcinoma. *Cancer Medicine*.

[19] Yi M., Li A., Zhou L., Chu Q., Luo S., Wu K. (2021). Immune signature-based risk stratification and prediction of immune checkpoint inhibitor’s efficacy for lung adenocarcinoma. *Cancer Immunology, Immunotherapy*.

[20] Zengin T., Önal-Süzek T. (2020). Analysis of genomic and transcriptomic variations as prognostic signature for lung adenocarcinoma. *BMC Bioinformatics*.

[21] Ren X., Kang B., Zhang Z. (2018). Understanding tumor ecosystems by single-cell sequencing: promises and limitations. *Genome Biology*.

[22] Liang J., Chen Z., Huang Y. (2022). Signatures of malignant cells and novel therapeutic targets revealed by single-cell sequencing in lung adenocarcinoma. *Cancer Medicine*.

[23] Newman A. M., Steen C. B., Liu C. L. (2019). Determining cell type abundance and expression from bulk tissues with digital cytometry. *Nature Biotechnology*.

[24] Schaum N., Lehallier B., Hahn O. (2020). Ageing hallmarks exhibit organ-specific temporal signatures. *Nature*.

[25] Liang L., Yu J., Li J. (2021). Integration of scRNA-seq and bulk RNA-seq to analyse the heterogeneity of ovarian cancer immune cells and establish a molecular risk model. *Frontiers in Oncology*.

[26] Qin G., Du L., Ma Y., Yin Y., Wang L. (2021). Gene biomarker prediction in glioma by integrating scRNA-seq data and gene regulatory network. *BMC Medical Genomics*.

[27] Zheng L., Li L., Xie J., Jin H., Zhu N. (2021). Six novel biomarkers for diagnosis and prognosis of esophageal squamous cell carcinoma: validated by scRNA-seq and qPCR. *Journal of Cancer*.

[28] Jerby-Arnon L., Shah P., Cuoco M. S. (2018). A cancer cell program promotes T cell exclusion and resistance to checkpoint blockade. *Cell*.

[29] Stankovic B., Bjørhovde H. A. K., Skarshaug R. (2019). Immune cell composition in human non-small cell lung cancer. *Frontiers in Immunology*.

[30] Fehniger T. A., Cooper M. A., Nuovo G. J. (2003). CD56^bright^ natural killer cells are present in human lymph nodes and are activated by T cell-derived IL-2: a potential new link between adaptive and innate immunity. *Blood*.

[31] Morvan M. G., Lanier L. L. (2016). NK cells and cancer: you can teach innate cells new tricks. *Nature Reviews Cancer*.

[32] Vivier E., Raulet D. H., Moretta A. (2011). Innate or adaptive immunity? The example of natural killer cells. *Science*.

[33] Pockley A. G., Vaupel P., Multhoff G. (2020). NK cell-based therapeutics for lung cancer. *Expert Opinion on Biological Therapy*.

[34] Pesce S., Greppi M., Grossi F. (2019). PD/1-PD-Ls checkpoint: insight on the potential role of NK cells. *Frontiers in Immunology*.

[35] Fu Y., Huang R., du J., Yang R., An N., Liang A. (2010). Glioma-derived mutations in _IDH_ : from mechanism to potential therapy. *Biochemical and Biophysical Research Communications*.

[36] Li J., He Y., Tan Z. (2018). Wild-type IDH2 promotes the Warburg effect and tumor growth through HIF1*α* in lung cancer. *Theranostics*.

[37] Moreno-Rubio J., Ponce S., Álvarez R. (2020). Clinical-pathological and molecular characterization of long-term survivors with advanced non-small cell lung cancer. *Cancer Biology & Medicine*.

[38] Nordgård O., Singh G., Solberg S. (2013). Novel molecular tumor cell markers in regional lymph nodes and blood samples from patients undergoing surgery for non-small cell lung cancer. *PLoS One*.

