# Supplementary material for: A Novel Prognostic Signature Revealed the Interaction of Immune Cells in Tumor Microenvironment Based on Single-Cell RNA Sequencing for Lung Adenocarcinoma
Source: J Immunol Res. 2022 Jul 1;2022:6555810. doi: 10.1155/2022/6555810 (PMC9270162; doi:10.1155/2022/6555810)
Supplement: Supplementary Materials — Figure S1: the cell annotation dot plot. The dot plot shows the percentage of clusters expressing marker genes (dot size) and the expression level (dot color). Figure S2: the GO and KEGG dot plot of brown module genes. The dot plot of KEGG (A) pathway and GO (B) enrichment terms. Figure S3: the summary and comparison of genomic analysis in the low- and high-risk groups. The low-risk (A) and high-risk groups (B) of patients' somatic mutation summary. The plots displayed the number of variants in each sample as a stacked bar plot and variant types as a boxplot summarized. Table S1: the cell annotation of 23 cell clusters. Table S2: the brown module's GO and KEGG enrichment analysis results. Table S3: the baseline characteristics between low- and high-risk groups. [file 6555810.f1.docx]

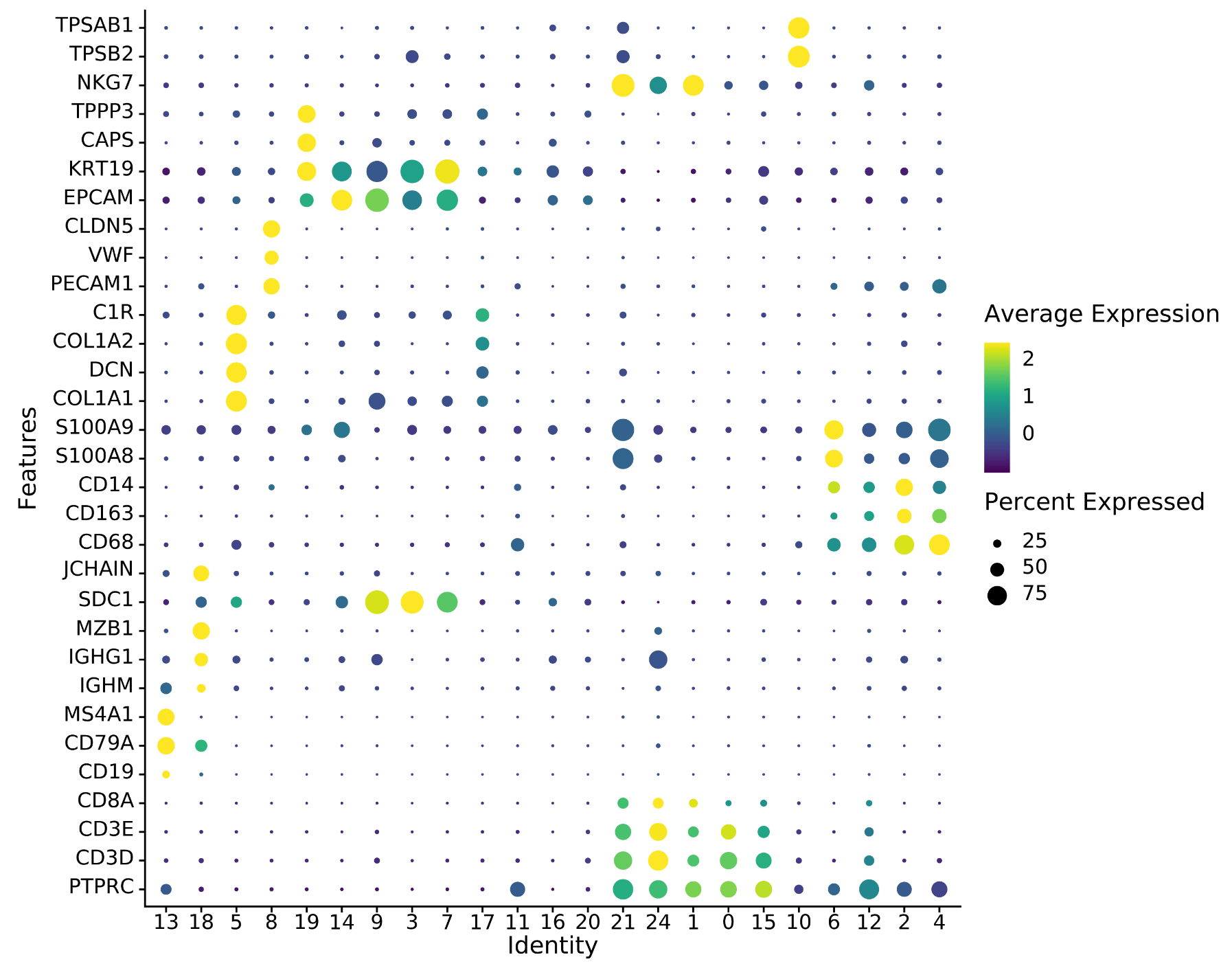
**Figure S1: The cell annotation dot plot**

The dot plot shows the percentage of clusters expressing marker genes (dot size) and the expression level (dot color).


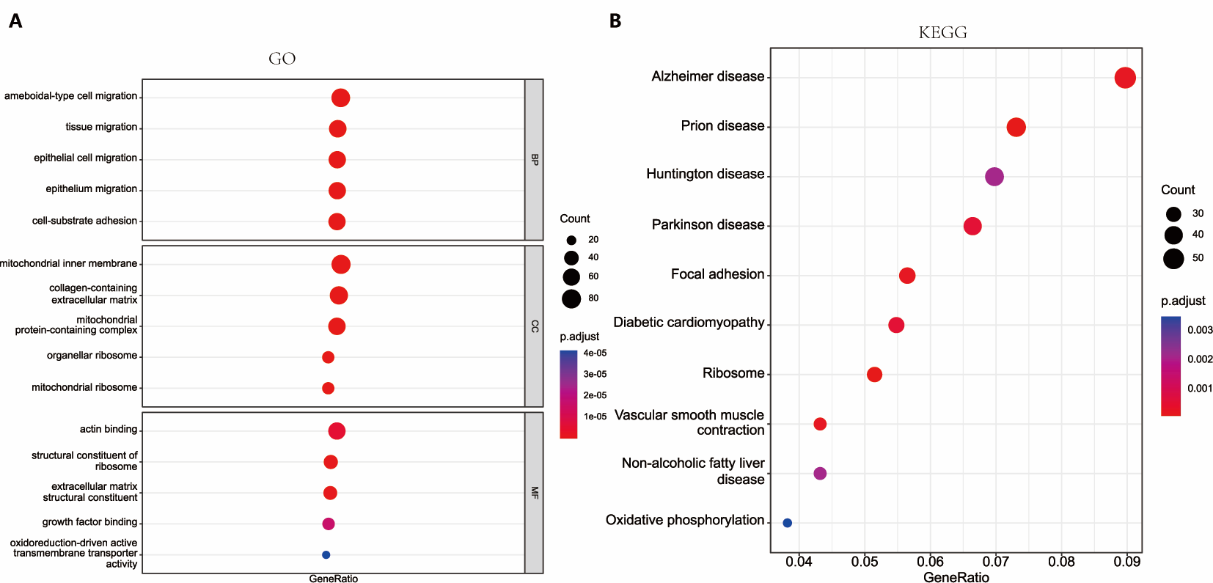


**Figure S2: the GO and KEGG dot plot of brown module’ genes**

The dot plot of KEGG(A) pathway and GO(B) enrichment terms

**Figure S3: The summary and comparison of genomic analysis in the low and high-risk group**

The low risk (A) and high-risk group (B) of patients' somatic mutation summary. The plots displayed the number of variants in each sample as a stacked bar plot and variant types as a boxplot summarized


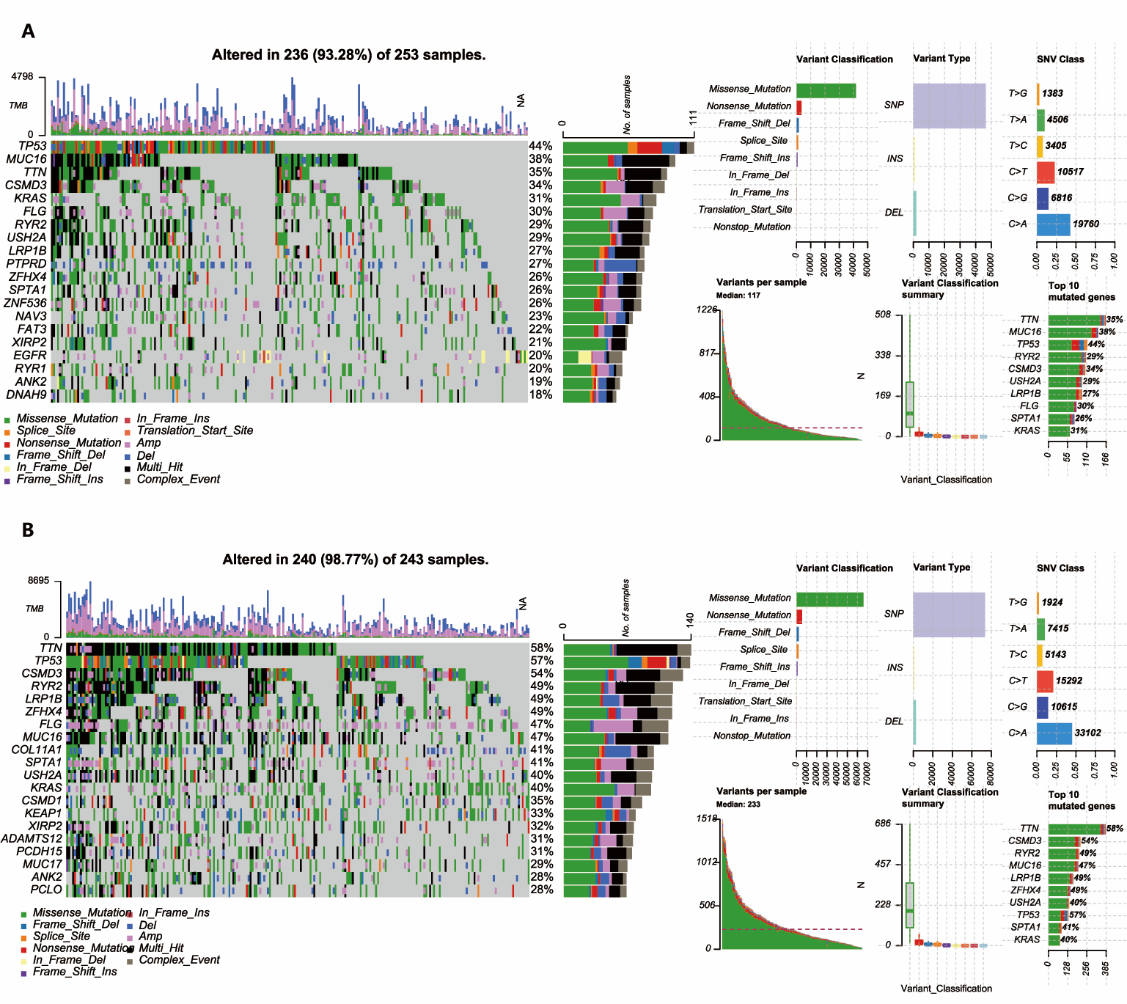


**Table S1: The cell annotation of 23 cell clusters.**

| **ClusterID** | **celltype** | **type** | **scores** | **ncells** | **celltype** | **marker** |
| --- | --- | --- | --- | --- | --- | --- |
| 0 | T_cells | Memory CD4+ T cells | 71952.58 | 47808 | T cells | CD3D,CD3E |
| 1 | NK_cell | CD8+ NKT-like cells | 86293.87 | 30779 | NK/NKT_cells | NKG7 |
| 2 | Macrophage | Macrophages | 70493.93 | 21410 | Macrophages | CD68,CD163 |
| 3 | Epithelial_cells | Cancer cells | 4391.665 | 3484 | Cancer | EPCAM,KRT19 |
| 4 | Macrophage | Macrophages | 55336.14 | 18194 | Macrophages | CD68,CD163 |
| 5 | Tissue_stem_cells | Platelets | 11164.56 | 13354 | Fibroblast | COL1A2,DCN,COL1A1,C1R |
| 6 | Monocyte | Neutrophils | 25485.89 | 10764 | Myeloid_cells | S100A8,S100A9 |
| 7 | Epithelial_cells | Cancer cells | 2808.513 | 1880 | Cancer | EPCAM,KRT19 |
| 8 | Endothelial_cells | Endothelial | 35226.44 | 8606 | Endothelial | PECAM1,VWF,CLDN5, |
| 9 | Epithelial_cells | Cancer cells | 1983.023 | 1443 | Cancer | EPCAM,KRT19 |
| 10 | NK_cell | Basophils | 53229.01 | 7716 | Mast_cell | TPSB2,TPSAB1 |
| 11 | DC | Myeloid Dendritic cells | 15352.27 | 6646 | Myeloid_cells | S100A8,S100A9 |
| 12 | Macrophage | Macrophages | 7067.584 | 5910 | Macrophages | CD68,CD163 |
| 13 | B_cell | Naive B cells | 19080.96 | 5215 | B_cells | CD79A,CD79B,IGHM,MS4A1 |
| 14 | Epithelial_cells | Cancer cells | 1800.099 | 1127 | Epithelial_cells | EPCAM,KRT19 |
| 15 | T_cells | Effector CD4+ T cells | 4589.285 | 4568 | T cells | CD3D,CD3E |
| 15 | T_cells | γδ-T cells | 4589.285 | 4568 | T cells | CD3D,CD3E |
| 16 | Epithelial_cells | Unknown | 14.29983 | 937 | Cancer | EPCAM,KRT19 |
| 17 | Tissue_stem_cells | HSC/MPP cells | 3093.627 | 2916 | Fibroblast | COL1A2,DCN,COL1A1,C1R |
| 18 | B_cell | Plasma B cells | 9062.287 | 2876 | Plasma B cells | IGHG1,MZB1,SDC1,JCHAIN |
| 19 | Epithelial_cells | Eosinophils | 5407.496 | 2831 | Ciliated_cells | CAPS,TPPP3 |
| 20 | Epithelial_cells | Unknown | 276.2224 | 2666 | Epithelial_cells | EPCAM,KRT19 |
| 21 | NK_cell | CD8+ NKT-like cells | 6981.02 | 1944 | NK/NKT_cells | NKG7 |
| 24 | T_cells | Naive CD8+ T cells | 2256.405 | 1083 | Naive CD8+ T cells | CD8A |

**Table S2: The brown module's GO and KEGG enrichment analysis results.**

| **module** | **GOID** | **term name** | **p-val** | **Bonf** | **FDR** | **size** |
| --- | --- | --- | --- | --- | --- | --- |
| brown | GO:0003674 | molecular_function | 2.50E-22 | 4.60E-17 | 2.30E-19 | 1337 |
| brown | GO:0098798 | mitochondrial protein-containing complex | 2.90E-21 | 5.30E-16 | 2.40E-18 | 1337 |
| brown | GO:0001944 | vasculature development | 2.10E-18 | 3.80E-13 | 1.30E-15 | 1337 |
| brown | GO:0072359 | circulatory system development | 2.20E-18 | 4.10E-13 | 1.40E-15 | 1337 |
| brown | GO:0001568 | blood vessel development | 1.60E-17 | 2.90E-12 | 9.00E-15 | 1337 |
| brown | GO:0005575 | cellular_component | 1.80E-17 | 3.30E-12 | 1.00E-14 | 1337 |
| brown | GO:0110165 | cellular anatomical entity | 1.30E-16 | 2.40E-11 | 6.70E-14 | 1337 |
| brown | GO:0005743 | mitochondrial inner membrane | 1.40E-16 | 2.60E-11 | 7.20E-14 | 1337 |
| brown | GO:0048514 | blood vessel morphogenesis | 2.20E-16 | 4.00E-11 | 1.10E-13 | 1337 |
| brown | GO:0035295 | tube development | 6.40E-16 | 1.20E-10 | 3.00E-13 | 1337 |
| brown | GO:0005488 | binding | 8.00E-16 | 1.50E-10 | 3.70E-13 | 1337 |
| brown | GO:0009987 | cellular process | 1.50E-15 | 2.80E-10 | 7.00E-13 | 1337 |
| brown | GO:0035239 | tube morphogenesis | 5.10E-15 | 9.30E-10 | 2.20E-12 | 1337 |
| brown | GO:0005740 | mitochondrial envelope | 9.30E-15 | 1.70E-09 | 3.80E-12 | 1337 |
| brown | GO:0008150 | biological_process | 1.20E-14 | 2.20E-09 | 4.80E-12 | 1337 |
| brown | GO:0019866 | organelle inner membrane | 1.40E-14 | 2.60E-09 | 5.70E-12 | 1337 |
| brown | GO:0031966 | mitochondrial membrane | 7.30E-14 | 1.30E-08 | 2.70E-11 | 1337 |
| brown | GO:0001525 | angiogenesis | 7.80E-14 | 1.40E-08 | 2.90E-11 | 1337 |
| brown | GO:0009653 | anatomical structure morphogenesis | 8.30E-13 | 1.50E-07 | 2.70E-10 | 1337 |
| brown | GO:0005739 | mitochondrion | 8.70E-13 | 1.60E-07 | 2.80E-10 | 1337 |
| brown | GO:0005515 | protein binding | 7.20E-12 | 1.30E-06 | 2.10E-09 | 1337 |
| brown | GO:0048731 | system development | 2.50E-11 | 4.50E-06 | 6.80E-09 | 1337 |
| brown | GO:0098800 | inner mitochondrial membrane protein complex | 3.20E-11 | 5.90E-06 | 8.60E-09 | 1337 |
| brown | GO:0000313 | organellar ribosome | 3.30E-11 | 6.00E-06 | 8.80E-09 | 1337 |
| brown | GO:0005761 | mitochondrial ribosome | 3.30E-11 | 6.00E-06 | 8.80E-09 | 1337 |
| brown | GO:0032502 | developmental process | 5.60E-11 | 1.00E-05 | 1.50E-08 | 1337 |
| brown | GO:0048646 | anatomical structure formation involved in morphogenesis | 6.60E-11 | 1.20E-05 | 1.70E-08 | 1337 |
| brown | GO:0031967 | organelle envelope | 9.30E-11 | 1.70E-05 | 2.40E-08 | 1337 |
| brown | GO:0031975 | envelope | 9.30E-11 | 1.70E-05 | 2.40E-08 | 1337 |
| brown | GO:0048856 | anatomical structure development | 1.40E-10 | 2.60E-05 | 3.60E-08 | 1337 |
| brown | GO:0061061 | muscle structure development | 1.50E-10 | 2.80E-05 | 3.80E-08 | 1337 |
| brown | GO:0007275 | multicellular organism development | 2.00E-10 | 3.70E-05 | 4.90E-08 | 1337 |
| brown | GO:0048869 | cellular developmental process | 5.20E-10 | 9.60E-05 | 1.20E-07 | 1337 |

| module | ID | term name | p-val | Bonf | FDR | size |
| --- | --- | --- | --- | --- | --- | --- |
| brown | MSigDB.M39114 | AIZARANI_LIVER_C10_MVECS_1 (MSigDB) | 1.70E-63 | 8.70E-59 | 1.20E-60 | 1337 |
| brown | MSigDB.M39167 | GAO_LARGE_INTESTINE_ADULT_CJ_IMMUNE_CELLS (MSigDB) | 2.50E-49 | 1.30E-44 | 1.20E-46 | 1337 |
| brown | MSigDB.M39128 | AIZARANI_LIVER_C29_MVECS_2 (MSigDB) | 1.30E-40 | 6.60E-36 | 4.40E-38 | 1337 |
| brown | MSigDB.M39049 | MANNO_MIDBRAIN_NEUROTYPES_HENDO (MSigDB) | 4.70E-33 | 2.50E-28 | 1.20E-30 | 1337 |
| brown | MSigDB.M40158 | DESCARTES_FETAL_CEREBELLUM_VASCULAR_ENDOTHELIAL_CELLS (MSigDB) | 1.70E-30 | 8.90E-26 | 4.00E-28 | 1337 |
| brown | MSigDB.M39176 | MURARO_PANCREAS_ENDOTHELIAL_CELL (MSigDB) | 5.30E-30 | 2.80E-25 | 1.20E-27 | 1337 |
| brown | MSigDB.M39117 | AIZARANI_LIVER_C13_LSECS_2 (MSigDB) | 3.90E-29 | 2.00E-24 | 8.50E-27 | 1337 |
| brown | MSigDB.M39050 | MANNO_MIDBRAIN_NEUROTYPES_HPERIC (MSigDB) | 1.70E-28 | 8.90E-24 | 3.70E-26 | 1337 |
| brown | MSigDB.M39018 | FAN_EMBRYONIC_CTX_BIG_GROUPS_BRAIN_ENDOTHELIAL (MSigDB) | 8.20E-28 | 4.30E-23 | 1.70E-25 | 1337 |
| brown | MSigDB.M41676 | TRAVAGLINI_LUNG_ALVEOLAR_FIBROBLAST_CELL (MSigDB) | 1.00E-27 | 5.20E-23 | 2.10E-25 | 1337 |
| brown | MSigDB.M39039 | FAN_EMBRYONIC_CTX_BRAIN_ENDOTHELIAL_1 (MSigDB) | 1.50E-24 | 7.60E-20 | 2.70E-22 | 1337 |
| brown | MSigDB.M40316 | DESCARTES_FETAL_THYMUS_VASCULAR_ENDOTHELIAL_CELLS (MSigDB) | 1.60E-24 | 8.20E-20 | 2.90E-22 | 1337 |
| brown | MSigDB.M40152 | DESCARTES_FETAL_ADRENAL_VASCULAR_ENDOTHELIAL_CELLS (MSigDB) | 8.90E-24 | 4.60E-19 | 1.60E-21 | 1337 |
| brown | MSigDB.M40189 | DESCARTES_FETAL_EYE_VASCULAR_ENDOTHELIAL_CELLS (MSigDB) | 2.70E-23 | 1.40E-18 | 4.50E-21 | 1337 |
| brown | MSigDB.M40167 | DESCARTES_FETAL_CEREBRUM_VASCULAR_ENDOTHELIAL_CELLS (MSigDB) | 3.90E-23 | 2.00E-18 | 6.60E-21 | 1337 |
| brown | MSigDB.M41659 | TRAVAGLINI_LUNG_ALVEOLAR_EPITHELIAL_TYPE_1_CELL (MSigDB) | 4.70E-23 | 2.40E-18 | 7.80E-21 | 1337 |
| brown | MSigDB.M39301 | CUI_DEVELOPING_HEART_C4_ENDOTHELIAL_CELL (MSigDB) | 2.20E-22 | 1.10E-17 | 3.50E-20 | 1337 |
| brown | MSigDB.M40283 | DESCARTES_FETAL_PLACENTA_VASCULAR_ENDOTHELIAL_CELLS (MSigDB) | 9.50E-22 | 4.90E-17 | 1.50E-19 | 1337 |
| brown | MSigDB.M39277 | DURANTE_ADULT_OLFACTORY_NEUROEPITHELIUM_PERICYTES (MSigDB) | 4.00E-21 | 2.10E-16 | 6.10E-19 | 1337 |
| brown | MSigDB.M39113 | AIZARANI_LIVER_C9_LSECS_1 (MSigDB) | 1.30E-20 | 6.60E-16 | 1.90E-18 | 1337 |
| brown | MSigDB.M39209 | HAY_BONE_MARROW_STROMAL (MSigDB) | 1.90E-19 | 9.70E-15 | 2.60E-17 | 1337 |
| brown | MSigDB.M41743 | RUBENSTEIN_SKELETAL_MUSCLE_ENDOTHELIAL_CELLS (MSigDB) | 1.20E-18 | 6.00E-14 | 1.50E-16 | 1337 |
| brown | MSigDB.M39040 | FAN_EMBRYONIC_CTX_BRAIN_ENDOTHELIAL_2 (MSigDB) | 1.20E-18 | 6.30E-14 | 1.60E-16 | 1337 |
| brown | MSigDB.M40230 | DESCARTES_FETAL_LIVER_VASCULAR_ENDOTHELIAL_CELLS (MSigDB) | 6.60E-18 | 3.40E-13 | 8.30E-16 | 1337 |
| brown | MSigDB.M41666 | TRAVAGLINI_LUNG_CAPILLARY_INTERMEDIATE_1_CELL (MSigDB) | 1.40E-17 | 7.20E-13 | 1.70E-15 | 1337 |
| brown | MSigDB.M13273 | DELYS_THYROID_CANCER_DN (MSigDB) | 1.00E-16 | 5.30E-12 | 1.20E-14 | 1337 |
| brown | MSigDB.M1578 | BOQUEST_STEM_CELL_DN (MSigDB) | 3.10E-16 | 1.60E-11 | 3.70E-14 | 1337 |
| brown | MSigDB.M39241 | LAKE_ADULT_KIDNEY_C22_ENDOTHELIAL_CELLS_GLOMERULAR_CAPILLARIES (MSigDB) | 3.50E-16 | 1.80E-11 | 4.10E-14 | 1337 |
| brown | MSigDB.M39153 | GAO_LARGE_INTESTINE_24W_C2_MKI67POS_PROGENITOR (MSigDB) | 6.80E-16 | 3.60E-11 | 7.90E-14 | 1337 |
| brown | MSigDB.M41746 | RUBENSTEIN_SKELETAL_MUSCLE_SMOOTH_MUSCLE_CELLS (MSigDB) | 7.60E-16 | 4.00E-11 | 8.80E-14 | 1337 |
| brown | MSigDB.M40215 | DESCARTES_FETAL_INTESTINE_VASCULAR_ENDOTHELIAL_CELLS (MSigDB) | 2.30E-15 | 1.20E-10 | 2.60E-13 | 1337 |
| brown | MSigDB.M19391 | LIU_PROSTATE_CANCER_DN (MSigDB) | 2.40E-15 | 1.20E-10 | 2.70E-13 | 1337 |
| brown | MSigDB.M39246 | LAKE_ADULT_KIDNEY_C27_VASCULAR_SMOOTH_MUSCLE_CELLS_AND_PERICYTES (MSigDB) | 4.90E-15 | 2.50E-10 | 5.30E-13 | 1337 |
| brown | MSigDB.M14791 | SABATES_COLORECTAL_ADENOMA_DN (MSigDB) | 1.40E-14 | 7.40E-10 | 1.50E-12 | 1337 |
| brown | MSigDB.M39121 | AIZARANI_LIVER_C20_LSECS_3 (MSigDB) | 3.30E-14 | 1.70E-09 | 3.40E-12 | 1337 |
| brown | MSigDB.M39243 | LAKE_ADULT_KIDNEY_C24_ENDOTHELIAL_CELLS_AEA_AND_DVR (MSigDB) | 1.70E-13 | 8.80E-09 | 1.70E-11 | 1337 |
| brown | MSigDB.M39245 | LAKE_ADULT_KIDNEY_C26_MESANGIAL_CELLS (MSigDB) | 1.80E-13 | 9.30E-09 | 1.70E-11 | 1337 |
| brown | MSigDB.M41710 | FAN_OVARY_CL8_MATURE_CUMULUS_GRANULOSA_CELL_2 (MSigDB) | 3.70E-13 | 1.90E-08 | 3.50E-11 | 1337 |
| brown | MSigDB.M39122 | AIZARANI_LIVER_C21_STELLATE_CELLS_1 (MSigDB) | 5.70E-13 | 3.00E-08 | 5.30E-11 | 1337 |
| brown | MSigDB.M39279 | DURANTE_ADULT_OLFACTORY_NEUROEPITHELIUM_VASCULAR_SMOOTH_MUSCLE_CELLS (MSigDB) | 8.20E-13 | 4.20E-08 | 7.50E-11 | 1337 |
| brown | MSigDB.M15112 | WONG_MITOCHONDRIA_GENE_MODULE (MSigDB) | 2.40E-12 | 1.20E-07 | 2.10E-10 | 1337 |
| brown | MSigDB.M39264 | HU_FETAL_RETINA_FIBROBLAST (MSigDB) | 2.70E-12 | 1.40E-07 | 2.30E-10 | 1337 |
| brown | MSigDB.M40266 | DESCARTES_FETAL_PANCREAS_VASCULAR_ENDOTHELIAL_CELLS (MSigDB) | 4.50E-12 | 2.30E-07 | 3.80E-10 | 1337 |
| brown | MSigDB.M27446 | REACTOME_MITOCHONDRIAL_TRANSLATION (MSigDB) | 4.50E-12 | 2.40E-07 | 3.90E-10 | 1337 |
| brown | MSigDB.M41712 | FAN_OVARY_CL10_PUTATIVE_EARLY_ATRESIA_GRANULOSA_CELL (MSigDB) | 4.60E-12 | 2.40E-07 | 3.90E-10 | 1337 |
| brown | MSigDB.M41669 | TRAVAGLINI_LUNG_BRONCHIAL_VESSEL_2_CELL (MSigDB) | 5.90E-12 | 3.10E-07 | 5.00E-10 | 1337 |
| brown | MSigDB.M42508 | HOUNKPE_HOUSEKEEPING_GENES (MSigDB) | 6.40E-12 | 3.30E-07 | 5.40E-10 | 1337 |
| brown | MSigDB.M10605 | BERTUCCI_MEDULLARY_VS_DUCTAL_BREAST_CANCER_DN (MSigDB) | 8.60E-12 | 4.50E-07 | 7.20E-10 | 1337 |
| brown | MSigDB.M39131 | AIZARANI_LIVER_C32_MVECS_3 (MSigDB) | 2.10E-11 | 1.10E-06 | 1.70E-09 | 1337 |
| brown | MSigDB.M1451 | NAKAYAMA_SOFT_TISSUE_TUMORS_PCA2_DN (MSigDB) | 4.90E-11 | 2.60E-06 | 3.90E-09 | 1337 |
| brown | MSigDB.M40309 | DESCARTES_FETAL_STOMACH_VASCULAR_ENDOTHELIAL_CELLS (MSigDB) | 6.30E-11 | 3.30E-06 | 4.90E-09 | 1337 |
| brown | MSigDB.M39221 | LAKE_ADULT_KIDNEY_C2_PODOCYTES (MSigDB) | 7.40E-11 | 3.90E-06 | 5.70E-09 | 1337 |
| brown | MSigDB.M3837 | WEST_ADRENOCORTICAL_TUMOR_DN (MSigDB) | 9.20E-11 | 4.80E-06 | 7.10E-09 | 1337 |
| brown | MSigDB.M12176 | CAIRO_HEPATOBLASTOMA_CLASSES_DN (MSigDB) | 1.20E-10 | 6.40E-06 | 9.40E-09 | 1337 |
| brown | MSigDB.M1255 | INAMURA_LUNG_CANCER_SCC_DN (MSigDB) | 2.20E-10 | 1.10E-05 | 1.60E-08 | 1337 |
| brown | MSigDB.M41716 | FAN_OVARY_CL14_MATURE_SMOOTH_MUSCLE_CELL (MSigDB) | 3.40E-10 | 1.80E-05 | 2.50E-08 | 1337 |
| brown | MSigDB.M41678 | TRAVAGLINI_LUNG_PERICYTE_CELL (MSigDB) | 4.00E-10 | 2.10E-05 | 2.90E-08 | 1337 |
| brown | MSigDB.M14142 | BERTUCCI_INVASIVE_CARCINOMA_DUCTAL_VS_LOBULAR_DN (MSigDB) | 4.50E-10 | 2.40E-05 | 3.30E-08 | 1337 |
| brown | MSigDB.M40257 | DESCARTES_FETAL_MUSCLE_VASCULAR_ENDOTHELIAL_CELLS (MSigDB) | 7.20E-10 | 3.80E-05 | 5.10E-08 | 1337 |
| brown | MSigDB.M5936 | HALLMARK_OXIDATIVE_PHOSPHORYLATION (MSigDB) | 7.60E-10 | 3.90E-05 | 5.40E-08 | 1337 |
| brown | MSigDB.M17923 | BOQUEST_STEM_CELL_CULTURED_VS_FRESH_UP (MSigDB) | 1.20E-09 | 6.00E-05 | 8.00E-08 | 1337 |
| brown | MSigDB.M4950 | ACEVEDO_LIVER_TUMOR_VS_NORMAL_ADJACENT_TISSUE_UP (MSigDB) | 1.20E-09 | 6.30E-05 | 8.30E-08 | 1337 |
| brown | MSigDB.M39320 | CUI_DEVELOPING_HEART_CORONARY_VASCULAR_ENDOTHELIAL_CELL (MSigDB) | 1.30E-09 | 7.00E-05 | 9.20E-08 | 1337 |
| brown | MSigDB.M40151 | DESCARTES_FETAL_ADRENAL_STROMAL_CELLS (MSigDB) | 1.90E-09 | 9.60E-05 | 1.30E-07 | 1337 |

**Table S3: The baseline characteristics between low and high-risk group**

|  | **low** | **high** | **p.overall** | **N** |
| --- | --- | --- | --- | --- |
|  | ***N=243*** | ***N=258*** |  |  |
| Age | 67.0 [59.0;73.0] | 65.0 [58.0;72.0] | 0.179 | 491 |
| Age_group: |  |  | 0.227 | 491 |
| <=60 | 70 (29.2%) | 87 (34.7%) |  |  |
| >60 | 170 (70.8%) | 164 (65.3%) |  |  |
| Age_median: |  |  | 0.690 | 491 |
| <=66 | 119 (49.6%) | 130 (51.8%) |  |  |
| >66 | 121 (50.4%) | 121 (48.2%) |  |  |
| Gender: |  |  | 0.031 | 501 |
| female | 143 (58.8%) | 126 (48.8%) |  |  |
| male | 100 (41.2%) | 132 (51.2%) |  |  |
| Race: |  |  | 0.158 | 501 |
| white | 195 (80.2%) | 190 (73.6%) |  |  |
| black | 19 (7.82%) | 32 (12.4%) |  |  |
| other | 29 (11.9%) | 36 (14.0%) |  |  |
| Smoke: |  |  | 0.012 | 487 |
| NO | 112 (47.3%) | 89 (35.6%) |  |  |
| YES | 125 (52.7%) | 161 (64.4%) |  |  |
| Tstage: |  |  | <0.001 | 498 |
| T1 | 111 (45.9%) | 56 (21.9%) |  |  |
| T2 | 114 (47.1%) | 155 (60.5%) |  |  |
| T3 | 11 (4.55%) | 32 (12.5%) |  |  |
| T4 | 6 (2.48%) | 13 (5.08%) |  |  |
| Nstage: |  |  | 0.025 | 489 |
| N0 | 169 (71.9%) | 153 (60.2%) |  |  |
| N1 | 39 (16.6%) | 55 (21.7%) |  |  |
| N2 | 27 (11.5%) | 44 (17.3%) |  |  |
| N3 | 0 (0.00%) | 2 (0.79%) |  |  |
| Mstage: |  |  | 0.835 | 497 |
| M0 | 165 (68.5%) | 169 (66.0%) |  |  |
| M1 | 12 (4.98%) | 13 (5.08%) |  |  |
| MX | 64 (26.6%) | 74 (28.9%) |  |  |
| Stage_group: |  |  | 0.012 | 494 |
| early stage | 200 (83.3%) | 187 (73.6%) |  |  |
| later stage | 40 (16.7%) | 67 (26.4%) |  |  |
| Stage: |  |  | 0.002 | 494 |
| I | 151 (62.9%) | 118 (46.5%) |  |  |
| II | 49 (20.4%) | 69 (27.2%) |  |  |
| III | 28 (11.7%) | 53 (20.9%) |  |  |
| IV | 12 (5.00%) | 14 (5.51%) |  |  |
| Radiotherapy: |  |  | 0.069 | 417 |
| NO | 186 (89.4%) | 173 (82.8%) |  |  |
| YES | 22 (10.6%) | 36 (17.2%) |  |  |
| Tumor_site: |  |  | 0.513 | 501 |
| Lower lobe | 85 (35.0%) | 85 (32.9%) |  |  |
| Middle lobe | 8 (3.29%) | 13 (5.04%) |  |  |
| other site | 6 (2.47%) | 11 (4.26%) |  |  |
| Upper lobe | 144 (59.3%) | 149 (57.8%) |  |  |
